# Prevalence and features of ICF-disability in Spain as captured by the 2008 National Disability Survey

**DOI:** 10.1186/1471-2458-11-897

**Published:** 2011-11-28

**Authors:** Sarah Maierhofer, Javier Almazán-Isla, Enrique Alcalde-Cabero, Jesús de Pedro-Cuesta

**Affiliations:** 1 Department of Applied Epidemiology, National Centre of Epidemiology-Consortium for Biomedical Research in Neurodegenerative Diseases (Centro de Investigación Biomédica en Red sobre Enfermedades Neurodegenerativas - CIBERNED), Carlos III Institute of Health, Av. Monforte de Lemos 5, 28029 Madrid, Spain

**Keywords:** ADL, Disability, Epidemiology, ICF, Prevalence, Survey, WHO

## Abstract

**Background:**

Since 1986, the study of disability in Spain has been mainly addressed by National Disability Surveys (NDSs). While international attempts to frame NDS designs within the International Classification of Functioning, Disability and Health (ICF) have progressed, in general, the ICF has hardly been used in either the NDS or epidemiological studies. This study sought to identify ICF Activity- and Participation-related content in the most recent Spanish NDS, the 2008 Survey on Disabilities, Independence and Dependency Situations (*Encuesta sobre discapacidades, autonomía personal y situaciones de Dependencia *- *EDAD 2008*), and estimate the prevalence of such ICF-framed disability.

**Methods:**

*EDAD 2008 *methods and questions were perused. Of the 51 EDAD items analysed, 29 were backcoded to specific d2-d7 domains of the ICF Checklist and, by rating the recorded difficulty to perform specific tasks with or without help, these were then taken as performance and capacity respectively. A global ICF score was also derived, albeit lacking data for d1, "Learning and applying knowledge", d8, "Major Life Areas" and d9, "Community, Social and Civic Life". Data were grouped by sex, age, residence and initial positive screening, and prevalence figures were calculated by disability level both for the general population, using the originally designed weights, and for the population that had screened positive to disability. Data for institutionalised persons were processed separately.

**Results:**

Crude prevalence of ICF severe/complete and moderate disability among the community-dwelling population aged ≥6 years was 0.9%-2.2% respectively, and that of severe/complete disability among persons living in sheltered accommodation was 0.3%.

Prevalence of severe/complete disability was: higher in women than in men, 0.8% vs. 0.4%; increased with age; and was particularly high in domains such as "Domestic Life", 3.4%, "Mobility", 1.8%, and "Self-care", 1.9%, in which prevalence decreased when measured by reference to performance. Moreover, global scores indicated that severe/complete disability in these same domains was frequent among the moderately disabled group.

**Conclusions:**

The *EDAD 2008 *affords an insufficient data set to be ICF-framed when it comes to the Activity and Participation domains. Notwithstanding their unknown validity, ratings for available ICF domains may, however, be suitable for consideration under the ADL model of functional dependency, suggesting that there are approximately 500,000 persons suffering from severe/complete disability and 1,000,000 suffering from moderate disability, with half the latter being severely disabled in domains capable of benefiting from technical or personal aid. Application of EDAD data to the planning of services for regions and other subpopulations means that need for personal help must be assessed, unmet needs ascertained, and knowledge of social participation and support, particularly for the mentally ill, improved. International, WHO-supported co-operation in ICF planning and use of NDSs in Spain and other countries is needed.

## Introduction

The World Health Organization (WHO) approved the International Classification of Functioning, Disability and Health (ICF) in 2001 as a framework and classification for understanding the impact of health conditions on functioning and disability [1]. The ICF conceptual framework encompasses different components, such as body functions, body structures, activities and participation, with some personal and environmental factors being included among the causes of disability [2]. The ICF enables individuals' functioning and disability to be comprehensively described and categorised in a way that is systematic, standardised and readily understandable by all health professionals and social workers involved in the care and support of patients [2]. The ICF is increasingly used in different sectors, including health, social affairs, labour and education [3,4].

Conceivably, the most powerful and regularly used tools for describing how disability affects citizens and supporting social policies are National Disability Surveys (NDSs). Early efforts by the Disability Tabulations (DISTAB) group, including rigorous selection of questions from five NDSs and backcoding these to the ICF, suggested that cross-country comparisons would be restricted to comparable backcoded questions, and that the best basis for international comparability would thus be for questions' focus and format to be structured to the ICF during the surveys' development phase [5]. Mulhorn and Threats, using the ICF, described several-fold variations in age- and sex-specific prevalence of speech and hearing limitations across five countries included in the DISTAB study, and revealed difficulties in comparing figures based on measures of differing sensitivity [6]. Recently, international action to understand disability in populations was led by the Washington City Group (a working group of the United Nations) [7]. Using the ICF to develop the Irish National Disability Survey resulted in a broader range of disabilities encompassed and the incorporation of policy-relevant environmental factors, and prompted a discussion on ethics [8].

While it would seem that the ICF capacity and performance qualifiers are considered useful by rehabilitation and social workers [9], the use of capacity and performance prevalence data for assessing or developing social services and policies appears to have been less explored. Stineman et al, after confirmatory factor analysis of a large US population, proposed that a standard set of ICF core chapters -mobility, self-care and domestic life- could help link and co-ordinate care across general practitioners, rehabilitation professionals and social services, as well as the acute and long-term care sectors [10]. In Spain, a personal disability index has been proposed, based on unselected disabilities registered in the 1999 NDS and taking into account the severity and number of hours of personal help received per week [11].

The type of disability data collected among the Spanish population has changed over the years. National and certain regional health surveys contain specific information on impairments, limitations and restrictions assessed using patient-reported outcomes, such as the EQ-5, as well as time received from caregivers [12]. The large-scale, nation-wide household surveys conducted by trained interviewers in 1986 and 1999 (http:// http://www.ine.es/) were developed using the framework of the International Classification of Impairments, Disabilities and Handicaps (ICIDH). The most recent of these was the 2008 Survey on Disabilities, Independence and Dependency Situations (*Encuesta sobre discapacidades, autonomía personal y situaciones de Dependencia *- *EDAD 2008*) [13-15], which partly incorporated the ICF's understanding of disability and some of its concepts.

Accordingly, this study sought: (a) to explore the potential of *EDAD 2008 *data for an ICF-based interpretation, detect any ICF-biased aspects and implement a specific application of the *EDAD 2008 *after ICF re-structuring; (b) to quantify prevalence of disability for selected ICF domains and for an individual ICF global score; and, (c) to describe severity patterns among the disabled population.

## Methods

### The *EDAD 2008*

#### Study population and sampling methodology

The study population covered by the *EDAD 2008 *[16,17] consisted of two residence-based population samples, one community-dwelling and the other institutionalised. For the community-dwelling sample, a two-step, stratified, random sample was used to provide a representative sample for each province: firstly, a sample of census sections was drawn, and a sample of family dwellings was then randomly drawn within each section. Thereafter, all households within the dwelling were group screened, with one member of each household being interviewed as the main informant [16,17]. A total of 258,187 persons living in 91,290 households were thus screened for disability.

In the case of institutions, a sample of Spanish residential centres and hospitals, representative of the country's Autonomous Regions, was drawn [16,17]. The centres had been classified into two different types, namely: (a) home or residential centres; and (b) psychiatric or geriatric hospitals or centres for disabled persons. At each centre, a random sample of inhabitants was drawn, yielding a sample of 10,567 institutionalised persons.

#### Individually tailored data-collection

For screening purposes, a catalogue of 44 questions about possible "disabilities" read *verbatim*, was presented to the main informant of each household. It addressed the following eight domains: vision; audition; communication; learning and application of knowledge and performance of tasks; mobility; self-care; domestic life; interaction and interpersonal relationships [16-19]. Persons for whom at least one of these questions was answered in the positive were then examined in depth, using a detailed disability questionnaire, covering the above-mentioned eight domains, medical conditions, diagnoses, professional life, education, discrimination, social contacts, accessibility and main caregivers. For some items, the level of difficulty experienced in a given item was measured against two different backgrounds, namely, the difficulty encountered when performing without aid and that encountered when performing with technical or personal aid [18,19]. In addition, the technical or "*de facto*" personal help received was recorded. For subjects aged under 6 years, an adapted version of the questionnaire was used [18,19]. To evaluate the disabilities of children aged < 6 years, an informant was given a list of possible limitations and asked to select those that were present. All institutionalised persons allocated to the sample were interviewed with an adapted version of the detailed questionnaire used for the household-based disability study.

#### Database structure and records on study

The National Institute of Statistics (*Instituto Nacional de Estadística *- *INE*) provided us with individual *EDAD 2008 *records. Following the survey format, the data were organised into four data sets, i.e., one covering all persons screened in households (denoted as household data set), one covering persons who screened positive and answered the detailed questionnaire about their disabilities (disability data set), one covering positively screened children (data limitations), and lastly, one covering all institutionalised persons (data centres). To cover specific issues of relevance, we used data from the above-mentioned four data sets, selecting the most appropriate for any given purpose.

### ICF framing of *EDAD 2008 *data

#### ICF-EDAD 2008 cross-walking; ICF backcoding of selected EDAD 2008 variables

This study disregarded ICF components featured under "Body Structures" or "Body Functions" and the so-called "Contextual Factors", focused on ICF categories shown under the "Activity and Participation" section (corresponding to ICF codes beginning with the letter **d**) and, for cross-comparison purposes, selected those included in the ICF Checklist (http://www.who.int/classifications/icf/training/icfchecklist.pdf) [20]. An important reason for using the frame of the ICF Checklist rather than that of another instrument, such as the WHODAS-2, is that the former incorporates *capacity *and *performance *qualifiers. After examining ICF Checklist items one-by-one and initially selecting the most likely corresponding variables from the *EDAD 2008 *disability questionnaire, data collected by the *EDAD 2008 *were then imported to individual ICF Checklist records, as indicated in Table 1. As will be seen from this table, almost all domain-d2 to -d7 items included in the ICF checklist are represented in the *EDAD 2008*. However, appropriate information for domains d1, "Learning and applying knowledge", d8, "Major life areas", and d9, "Community, social and civic life", was not found, despite the fact that data on educational level, occupational status, monthly income, feeling of discrimination and spare-time activities were available in the *EDAD 2008*. Although the *EDAD 2008 *sometimes contains limited information on a subject's social activities, e.g., leisure-time reading, or job status, it does not indicate whether the person in question experiences any problems, i.e. restrictions, in performing such activities. In a second step, following review and reviewer-recommended external expert consultation, a number of variables were additionally dropped from those that had been initially selected. Consequently, the ICF Checklist frame resulting from the *EDAD 2008 *questionnaire was an incomplete version, since a considerably high number of codes were rejected for data import and left as empty codes after these two steps (see Table 1).

**Table 1:** *EDAD 2008 *items of potential interest and those finally selected for ICF backcoding*.

ICF Checklist variables	*EDAD 2008 *variables considered (regardless of selection)		*EDAD 2008 *item used	
**Short list of activity and participation domains**	**Variable**	**Content of *EDAD 2008 *variables**	**Capacity**	**Performance**

**d1. Learning and applying knowledge**				

**d110 **Watching	APR_14	*to hold a gaze or pay attention when listening*	14.2	14.2

**d115 **Listening	APR_14	*to hold a gaze or pay attention when listening*	14.2	14.2

**d140 **Learning to read	APR_15	*to learn to perform simple tasks*	15.2	15.2

**d145 **Learning to write	APR_15	*to learn to perform simple tasks*	15.2	15.2

**d150 **Learning to calculate	APR_15	*to learn to perform simple tasks*	15.2	15.2

**d175 **Solving problems				

**d2. General tasks and demands**				

**d210 **Undertaking a single task	APR_16*	to perform simple tasks	16.2	16.3.b^pt^

**d220 **Undertaking multiple tasks	APR_17*	to perform complex tasks	17.2	17.3.b^pt^

**d3. Communication**				

**d310 **Communicating with -- receiving -- spoken messages	COM_9*	to understand the meaning of what other persons say	9.2	9.3.b^p^

**d315 **Communicating with -- receiving -- non-verbal messages	COM_11*	to understand and express yourself via gestures, symbols, illustrations or sounds	11.2	11.2

**d330 **Speaking	COM_8*	to speak intelligibly or utter coherent phrases	8.2	8.3.b^t^

**d335 **Producing non-verbal messages				

**d350 **Conversation	COM_12*	to hold a dialogue and exchange ideas with one or more persons	12.2	12.2

**d4. Mobility**				

**d430 **Lifting and carrying objects	MOV_24*	to lift or carry objects	24.2	24.3.b^pt^

**d440 **Fine hand use	MOV_26*	to lift or carry small objects	26.2	26.3.b^pt^

**d450 **Walking	MOV_20*^m^	to walk and move inside the home	20.2	20.3.b^pt^
	
	MOV_21*^m^	to walk and move around the home	21.2	21.3.b^pt^

**d465 **Moving around using equipment				

**d470 **Using transportation	MOV_22*	to get around via passenger transport	22.2	22.3.b^pt^

**d475 **Driving	MOV_23*	to drive vehicles	23.2	23.3.b^t^

**d5. Self-Care**				

**d510 **Washing oneself	AUT_27*	to wash or dry different body parts	27.2	27.3.b^pt^

**d520 **Caring for body parts	AUT_28*	to perform basic grooming	28.2	28.3.b^pt^

**d530 **Toileting	AUT_29^max^	to carry out activities related to urination	29.2	29.3.b^pt^
	
	AUT_30*^max^	to carry out activities related to defecation	30.2	30.3.b^pt^
	
	AUT_31*^max^	to carry out activities related to menstrual care (only for women)	31.2	31.3.b^pt^

**d540 **Dressing	AUT_32*	to dress or undress	32.2	32.3.b^pt^

**d550 **Eating	AUT_33	*to eat and drink*	33.2	33.3.b^pt^

**d560 **Drinking	AUT_33	*to eat and drink*	33.2	33.3.b^pt^

**d570 **Looking after one's health	AUT_34*^max^	to follow medical prescriptions	34.2	34.3.b^pt^
	
	AUT_35*^max^	to avoid dangerous situations	35.2	35.3.b^pt^

**d6. Domestic life**				

**d620 **Acquisition of goods and services	VDOM_36*	to do shopping	36.2	36.3.b^pt^

**d630 **Preparation of meals	VDOM_37*	to prepare meals	37.2	37.3.b^pt^

**d640 **Doing housework	VDOM_38*	to carry out housework	38.2	38.3.b^pt^

**d660 **Assisting others				

**d7. Interpersonal interactions and relationships**				

**d710 **Basic interpersonal interactions	INTER_39*	to show other persons affection, respect or transmit feelings	39.2	39.2

**d720 **Complex interpersonal interactions				

**d730 **Relating with strangers	INTER_40*	to relate to strangers	40.2	40.2

**d740 **Formal relationships	INTER_41*	to initiate and maintain relations with subordinates, peers or superiors	41.2	41.2

**d750 **Informal social relationships	INTER_42*	to initiate and maintain relations with friends, neighbours, acquaintances or colleagues	42.2	42.2

**d760 **Family relationships	INTER_43*	to initiate and maintain family relations	43.2	43.2

**d770 **Intimate relationships	INTER_44*	to initiate and maintain intimate or sexual relations	44.2	44.2

**d8. Major life areas**				

**d810 **Informal education **d820 **School education **d830 **Higher education	F_1	*Level of studies completed*		
	
	F_2	*In relation to your finished studies, what is your diploma degree or graduate degree?*		
	
	F_3	*In the last five years, have you done any vocational training course?*		
	
	G_1	*In relation to education and school integration, what was your situation last week?*		

**d850 **Remunerative employment	E_1	Due to the onset or worsening of your disability, have you had to amend your relationship with economic activity or your occupation?		
	
	E_5	Relation with economic activity in the past week.		
	
	E_6	*Have you worked at some point as an employee or freelance worker?*		
	
	E_15	Professional situation		
	
	E_20	*Why did you stop working?*		

**d860 **Basic economic transactions				

**d870 **Economic self-sufficiency	F_3^a^	*What is the monthly amount of the total income for this household?*		

**d9. Community, social and civic life**				

**d910 **Community Life	H_2_11	*In the last 12 months, have you felt discriminated against on the basis of your disability in any of the following situations? In social participation*		
	
	H_2_12	*In the last 12 months, have you felt discriminated against on the basis of your disability in any of the following situations? In social relations*		
	
	I_4_2	Have you had any opportunities in the last 12 months? Relating with friends or persons who are close		

**d920 **Recreation and leisure	I_5_2	*What activities do you spend most of your spare time on, and which would you like to carry out for enjoyment or recreation that you do not already, due to your disability? Please select the three main activities in both columns*.		

**d930 **Religion and spirituality				

**d940 **Human rights				

**d950 **Political life and citizenship	H_2_7	*In the last 12 months, have you felt discriminated against on the basis of your disability in any of the following situations? - Public Administration*		

Where applicable, the item for difficulty involved in activities undertaken with technical or personal aid was taken as *performance*. Variables not marked with an asterisk and having their content shown in italics were excluded from backcoding, either from an interim list by two authors acting in consensus, or after reviewers' comments and consideration by an ICF expert. Spaces left blank indicate no *EDAD 2008 *item was deemed appropriate for ICF coding. Domains totally devoid of items considered valid for backcoding (d1) or represented by single *EDAD 2008 *items (d8,d9) were dropped from further analysis and results

As the extra question for performance was not always asked, the columns entitled "capacity" and "performance" show the number of the item from which the information was taken.

^a^This item is taken from the household instead of the disability questionnaire.

^P ^= personal assistance,^t ^= external technical aid,^pt ^= personal assistance or technical aid.

When there was more than one item linked to an ICF category the mean^m ^or the maximum^max ^of these items was used.

In accordance with a recognised, optional interpretation of *capacity *and *performance *as relating to difficulties in the absence or presence of technical and personal help, we took advantage of the above information on difficulty encountered with and without technical or personal aid to derive firstly a measure of *capacity *and thence a measure of *performance *from the *EDAD 2008*, both recorded in each individual ICF-framed incomplete Checklist record. In practice, measurements were taken in such a way that, if the answer about receiving any type of aid (technical, personal or both) was positive, the level of *performance *was assessed. To sum up, in the case of selected variables (see Table 1), two ICF disability indices were obtained, one for *capacity *and the other for *performance*, using the same ICF score conversion procedure explained below. Domains for which there were no questions on aid (d1, "Learning and applying knowledge", d7 "Interpersonal interactions and relationships", and, in part, d3 "Communication", see Table 1) were excluded from this method of deriving *performance*. For items from other domains where there was confirmation that no aid had been received, information on capacity was used to impute the value of performance, assuming that capacity equalled performance in such cases. In the *EDAD 2008*, we were unable to identify collected data on barriers, whether physical or social, and facilitators (other than the above-mentioned "help") linked to disability items.

#### Calculation of a tentative ICF checklist-oriented individual score

In the *EDAD 2008*, level of disability is rated on an ordinal scale with the following four response options: no/mild difficulty (1); moderate difficulty (2); severe difficulty (3); and complete difficulty (4). In the ICF, each response option corresponds to a percentage interval of "Activity limitation" or "Restriction in Social Participation", represented by the corresponding "qualifier" in five intervals (see Table 2), e.g., severe difficulty ranges from 50% to 95%, and complete difficulty from 96% to 100%. Hence, in order to arrive at a reasonable calculation of the mean of several ICF items, these had to be rescaled according to their equivalent percentage values, e.g., the response option, "severe difficulty", was recoded as 72.5%. To obtain a score, the mean value of any given ICF-framed selected item was calculated, scaled from 0% to 100% and recoded as ICF option 1 to 4, based on the ICF percentage intervals shown in Table 2. ICF *capacity *and, when available, *performance *indices in single domains were thus obtained, and categorised into four (1-4) rather than five (0-4) levels.

**Table 2:** ICF categories: (a) as originally denoted and described in words, numerals, percentages and mid-point scores for each categorical percentage-span; and (b) as backcoded in this study.

ICF (in words)	ICF (in categories)	ICF (in percentage terms)	*EDAD 2008 *categories	Mid-point score
No difficulty	0	0%-4%	No or mild difficulty	12%
		
Mild difficulty	1	5%-24%.		

Moderate difficulty	2	25%-49%	Moderate difficulty	37%

Severe difficulty	3	50%-95%	Severe difficulty	72.5%

Complete difficulty	4	96%-100%	Complete difficulty	98%

Since the *EDAD 2008 *drew no distinction between 0 and 1, these two options were combined as 0%-24%, which matches a mid-point score of 12%

A global score was obtained for *capacity *measurements but not for *performance*. To calculate such a global score, a total of 32 variables were -not always unequivocally- selected to build 32 ICF categories from the ICF checklist (Tables 1 and 3). Answers of inapplicable and missing values were replaced by 1 (no or mild difficulty).

Score intervals were then selected as values corresponding to ordinal ICF qualifiers. Qualifiers for ICF items resulting from *EDAD 2008 *data were obtained by combining the scores for the selected *EDAD 2008 *variables, as indicated in Table 1, where "Learning and applying knowledge" (d1), "Major life areas" (d7) and "Community, social and civic life" (d9) were excluded, both for the remaining specific domains and for the global score which lacked data from the excluded domains. In practice, the effect of such exclusion on the method is to render global scores rather insensitive to restrictions in social participation. Moreover, the fact that disability data were obtained after screening positive for at least one of 44 disability items, means that the value 1 for no/mild difficulty described in Table 2 and denoted as N, most frequently corresponded to "mild" difficulty, i.e., mild disability, and for our purposes will therefore be referred to as the "mild disability group". Screened negative status was denoted as SN.

### Epidemiological measurements

#### Prevalence proportions

Prevalence proportions were calculated in the following three different contexts for particular requirements or purposes: a) data from the screened population, including both the community-dwelling and institutionalised samples, were used to describe crude prevalence of disability among the country's population; b) the screened community-dwelling population alone (i.e., excluding the institutionalised sample) furnished data for age-specific and age- and sex-adjusted measurements among the country's population and; c) proportions in the population that screened positive were used to describe ICF disability patterns by ICF domain. Calculation of age-specific or age-adjusted prevalence required exclusion of the institutionalised population, due to constraints relating to categorical changes in age data according to type of centre and Autonomous Region (see Table 3). However, since the severely/completely disabled were living in institutions, some figures selected by centre- and age-group were occasionally included in counts for the purposes of completeness. Prevalence figures had only two different denominators, namely: the general -sometimes age- and sex-specific- Spanish population; and the disabled screened positive population used to describe proportions affected by different severity levels.

**Table 3:** Different age groupings (1, 2 and 3) used in the sample of institutionalised persons, and the grouping used in this study to merge the information on age

Grouping 1 Age years	n	Grouping 2 Age years	n	Grouping 3 Age years	n	Grouping used Age years	n
6-64	20879	6-79	13084	6-64	23446	6-64	44325

65-79	48007			> 65	9165	≥65	227599

≥80	144805	≥80	25622				

NS	76815	NS	251800	NS	257895	NS	18582

NS = Not Specified

The groups differ by Autonomous Region and type of centre (type 1: home or residential centres, type 2 psychiatric or geriatric hospitals or centres for disabled persons), encompassing:

Grouping 1: type-1 centres and Autonomous Regions 1, 2, 7, 8, 9, 10, 11, 13, 16 or type-2 centres and Autonomous Regions 1, 5, 9.

Grouping 2: Type-1 centres and Autonomous Regions 4, 5, 6, 7, 12, 14, 15, 17.

Grouping 3: Type-2 centres and Autonomous Regions 2, 3, 6, 7, 8, 11, 12, 13, 14, 15, 16, 17.

Column "n" shows the number of persons in each age-group after the cases had been weighted. The age groups used were 6-64 and over-64 years (Grouping 3). Consequently, the 6-79 age group in grouping 2 and all cases with no information whatsoever on age are set to "missing".

In view of the fact that (a) sample probability was different for community-dwelling and institutionalised persons, with the former being tailored to provinces and the latter to Autonomous Regions, and that (b) result interpretability in terms of representativeness vis-à-vis the Spanish resident population was required, *EDAD 2008 *weighting was systematically applied to the numerators and denominators of the above-mentioned prevalence. After weighting, the subsamples were found to be accurately representative of the resident Spanish population. Our estimate of 45,031,810 community-dwelling and 290,506 institutionalised persons fitted well with the data reported on the survey web page [14]. Comparison with the National Institute of Statistics' data on the general population at 1 January 2008 [21] for each Autonomous Region yielded numbers 1%-3% higher than the numbers obtained by us from the *EDAD 2008 *data, except for a 5% difference in the case of Melilla and Ceuta, two relatively small cities located in Northern Africa.

## Results

### Descriptive statistics for items in single disability domains

The distribution of ICF categories and percentage of missing values in community-dwelling and institutionalised persons, after the sample aged ≥6 years had been excluded, are shown in Table 4. The sample ratio of institutionalised to community-dwelling subject who screened positive was 1:13. In general, missing values were far less frequent in the institutionalised sample. The proportions of missing values ranged from 2.3% to 43.6% for the community-dwelling sample and from 0% to 0.6% for the institutionalised sample, except for item d475, "Driving", for which the proportions of missing values were 51.7% and 72.7% in the respective samples.

**Table 4:** Percentages for ICF-Categories (No/mild difficulty, N; moderate difficulty, M; severe difficulty, S; complete difficulty, C) and subscores for each of the seven domains, as well as missing values (MV) in percentages.

		Community-dwelling persons					Institutionalised persons				
**ICF**	**ICF title**	**N**	**M**	**S**	**C**	**MV**	**N**	**M**	**S**	**C**	**MV**

d2	General tasks and demands	91.37	0.89	3.49	4.24	7.96	59.9	6.15	16.28	17.67	0

d210	Undertaking a single task	90.86	2.00	2.54	4.60	7.41	68.96	5.10	7.54	18.41	0

d220	Undertaking multiple tasks	85.73	3.14	3.63	7.50	2.32	54.59	7.34	8.83	29.24	0

d3	Communication	94.22	1.08	3.26	1.43	13.54	60.94	12.75	19.26	7.05	0.16

d310	Communicating with -- receiving -- spoken messages	89.45	4.08	4.20	2.26	9.07	67.53	8.20	14.24	10.04	0.02

d315	Communicating with -- receiving -- non-verbal messages	92.38	1.79	2.76	3.07	11.68	68.22	5.01	10.49	16.27	0.03

d330	Speaking	88.26	4.49	4.35	2.90	7.82	66.72	7.44	13.48	12.37	0.04

d350	Conversation	88.27	3.86	4.10	3.78	7.94	61.97	7.37	13.07	17.59	0.08

d4	Mobility	89.58	4.06	4.78	1.57	60.27	20.23	31.85	39.78	8.14	72.76

d430	Lifting and carrying objects	48.6	17.14	18.71	15.55	28.43	52.28	9.09	10.10	28.53	0.02

d440	Fine hand use	63.78	12.73	12.74	10.75	43.61	64.76	7.54	9.80	17.89	0.02

d450	Walking	60.38	7.27	21.38	10.97	40.73	35.76	12.98	29.06	22.21	0.25

d470	Using transportation	45.45	13.82	18.40	22.33	23.80	21.71	14.89	15.13	48.27	0.12

d475	Driving	75.55	3.90	2.97	17.58	51.65	23.47	1.79	4.52	70.22	72.66

d5	Self-care	96.39	2.42	0.76	0.44	45.00	21.16	25.23	31.75	21.86	96.67

d510	Washing oneself	61.29	10.77	11.81	16.13	14.08	29.14	19.22	13.85	37.79	0.04

d520	Caring for body parts	62.26	9.59	11.01	17.13	15.19	28.66	17.69	13.59	40.06	0.04

d530	Toileting	96.76	2.10	0.37	0.77	44.86	37.94	17.07	11.14	33.85	96.65

d540	Dressing	66.64	10.56	10.20	12.60	20.47	45.68	13.69	11.24	29.38	0

d570	Looking after one's health	76.71	3.46	4.26	15.58	30.79	32.07	13.03	11.60	43.30	0.46

d6	Domestic life	61.37	5.17	10.70	22.76	25.63	19.97	10.25	16.60	53.17	0.62

d620	Acquisition of goods and services	47.81	10.74	12.21	29.25	5.52	22.60	9.91	8.08	59.41	0.29

d630	Preparation of meals	60.00	7.29	7.24	25.46	22.85	22.39	8.74	7.10	61.78	0.15

d640	Doing housework	51.01	10.23	12.13	26.63	10.73	23.44	9.09	7.28	60.19	0.19

d7	Interpersonal inter-actions and relationships	95.16	0.68	2.60	1.56	11.50	45.14	29.49	18.69	6.68	1.05

d710	Basic interpersonal interactions	92.63	2.41	2.88	2.09	8.94	75.02	5.72	9.37	9.89	0.04

d730	Relating with strangers	88.55	2.4	3.36	5.68	5.16	60.16	6.52	10.11	23.21	0.10

d740	Formal relationships	90.49	1.82	2.37	5.32	6.95	75.61	6.21	7.90	10.28	0.11

d750	Informal social relationships	90.17	2.38	3.15	4.29	6.67	69.41	5.87	9.02	15.69	0.04

d760	Family relationships	90.04	1.82	2.8	5.34	6.54	75.43	5.74	8.61	10.21	0.17

d770	Intimate relationships	88.36	1.51	2.37	7.77	4.82	38.83	4.81	9.20	47.16	0.63

All numbers were calculated using individually weighted data

### Prevalence of disability by ICF capacity index

Assuming that all persons living in centres screened positive, prevalence of disability as seen from positive screening nation-wide (all levels included) was: 9.6% among persons aged ≥6 years; 2.2% among persons aged 0-5 years; 9% among community-dwelling persons aged ≥6 years; and 0.7% for institutionalised persons among the over-all population aged ≥6 years. Prevalence of severe or complete disability as defined by ICF qualifiers 3 and 4 from *capacity *scores for all persons aged ≥6 years was 1.2%, with 0.9% corresponding to those living in the community and 0.3% to institutionalised persons using the same population denominator. In other words, approximately one out of four severely/completely disabled persons was institutionalised.

**Figure 1:**
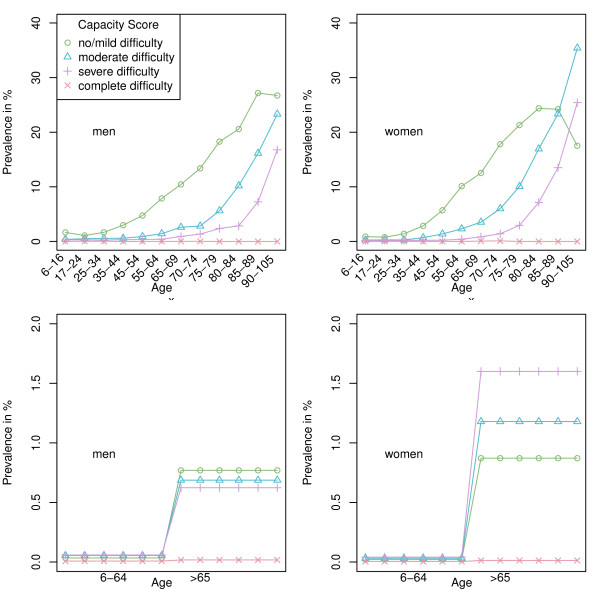
**Age- and sex-specific prevalence for four levels of total ICF disability score for capacity, separated into two components aged ≥6 years, i.e., community-dwelling screened positive residents (top) and institutionalised persons (bottom)**. Denominators for community-dwelling persons were obtained from the screened sample (community-dwelling persons); denominators for prevalence counts of institutionalised persons correspond to community-dwelling and institutionalised persons.

Figures 1a-d depict the age- and sex-specific prevalence of disability according to our ICF *capacity *index among the ≥6-year-old Spanish population separately by residential status, i.e., the community-dwelling and institutionalised components. It should be noted that screened negative persons are included in the denominators only, and that percentages do not therefore add up to 100. Age-specific disability prevalence among community-dwelling persons, Figures 1a and 1b, increased with age in men and women alike but was systematically higher among the latter, with crude prevalence of 7.3% in men and 10.6% in women. With regard to institutionalised persons, Figures 1c and 1d, in practice the proportion of institutionalised disabled persons per 100 inhabitants was restricted to the population aged < 65 years and ≥65 years and was approximately twice as high among women, particularly in the moderate and severely disabled levels. The crude prevalence of institutionalised disabled persons aged ≥65 years was 0.6% for men and 0.9% for women. The 0-5 year age group registered a 2.2% prevalence of disability. The paucity of information on type of limitation present meant that the ICF disability score could not be calculated for the 0-5 year age group.

**Figure 2:**
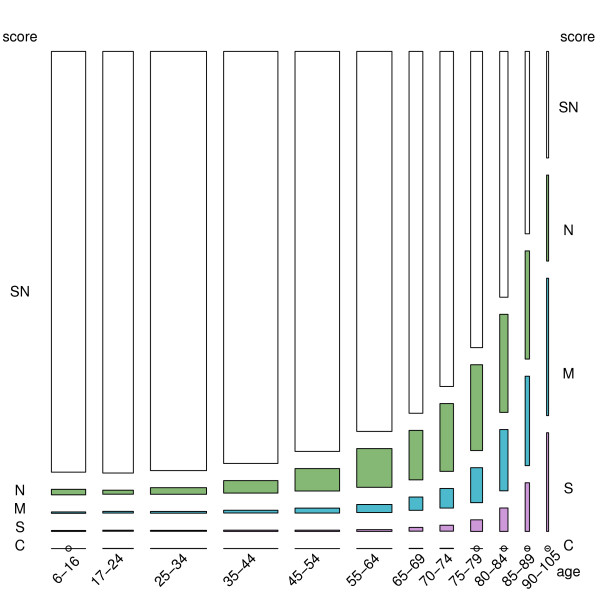
**Age-specific prevalence of different ICF disability levels, including disability-free/screened negative (SN) persons, as well as number of persons affected in each age and ICF group**. N = no/mild difficulty, M = moderate difficulty, S = severe difficulty, C = complete difficulty.

Figure 2 depicts the prevalence of ICF disability in the community-dwelling population (prevalence equals rectangle height), as well as the number of affected individuals of both sexes in each age category (numbers are equivalent to rectangle surface). Prevalence of disability increased with age, with the number of disabled persons decreasing among the very old. The highest prevalence corresponded to the moderately disabled with 920,310 persons, with the estimated total number of severely/completely disabled persons being 380,838. The highest numbers of severely/completely affected individuals were those aged ≥70 years. Numbers increased with age, i.e., rising from 26904 persons aged 70-74 years to 44137 persons aged 75-79 years, 61868 persons aged 80-84 years and 67125 persons aged 85-89 years, and then subsequently decreased to 62329 persons aged 90-105 years.

**Figure 3:**
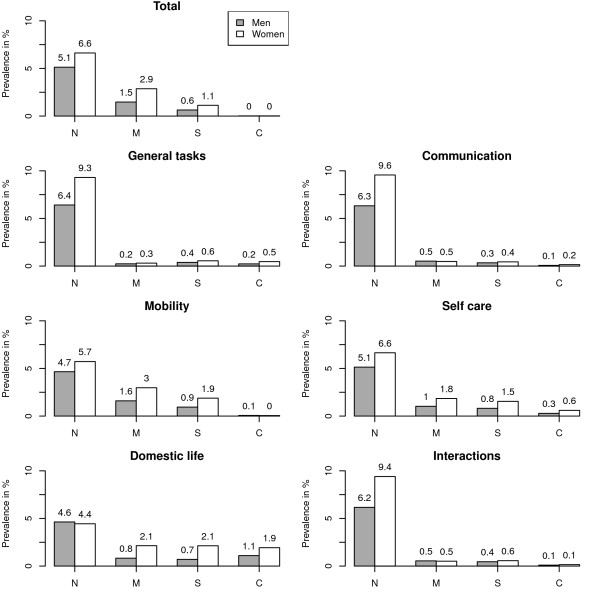
**Crude, sex- and domain-specific prevalence of different ICF severity scores among the general population from screened positive persons**. Percentages do not add up to 100% due to screened negative persons. N = no/mild difficulty, M = moderate difficulty, S = severe difficulty, C = complete difficulty.

Figure 3 depicts the prevalence of ICF *capacity*, both overall and for seven different ICF domains, as seen from the screened positive, community-dwelling population for each sex. Prevalence patterns varied, with the highest prevalence of severe/complete disability levels being 1.9% in "Mobility", 2.1% in "Self-care" and 4.1% in "Domestic life" for women (and 1%, 1.1% and 1.8% respectively for men). While the high prevalence patterns were similar across the sexes, differences between the sexes in magnitude were nevertheless present. In terms of total and "Mobility", "Self-care" and "Domestic life" domain scores, prevalence was higher among women than men. In contrast, differences between sexes were not substantial for prevalence of severe/complete disability in domains with lower prevalences, such as "General tasks and demands", "Communication" and "Interpersonal interactions and relationships".

### Capacity and performance patterns: ICF index by specific domain within single group by total ICF score

**Figure 4:**
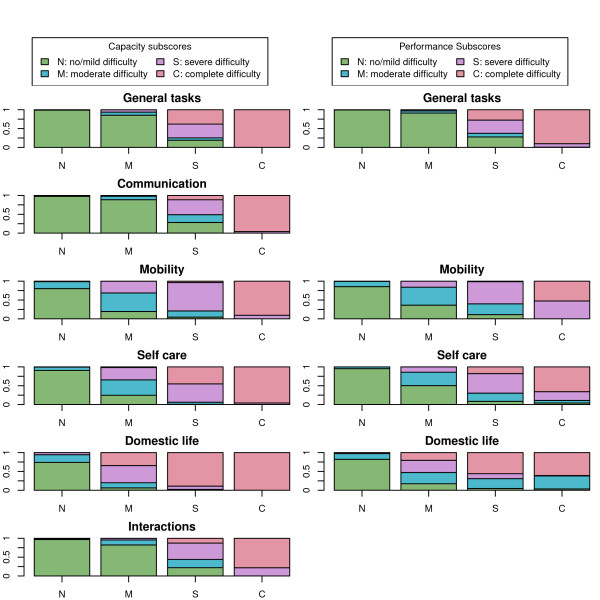
**Prevalence of domain- and qualifier-specific disability (by capacity or performance) among subgroups defined by total ICF capacity scores, for community-dwelling residents aged ≥6 years**. N = no/mild difficulty, M = moderate difficulty, S = severe difficulty, C = complete difficulty.

The distribution by disability level for *capacity *(left) and *performance *(right) subscores in the above-mentioned available ICF domains for groups of positively screened persons with four different total ICF capacity levels (N,M,S,C) is depicted in Figure 4. The most remarkable feature, more clearly visible in the case of *capacity *(left), was to be seen for groups of moderately disabled subjects in terms of total ICF score (M groups), in that these registered considerable proportions of persons with severe or complete *capacity *subscores, particularly in "Domestic life", 80%, "Mobility", 31.3% and "Self-care", 34.5%, with severe disability in these domains thus being relatively masked by the total ICF score. A second aspect of differences between *capacity *(left) and *performance *(right) patterns, once again more clearly visible for the moderately disabled by total ICF score, was the relative invariance in *capacity *versus *performance *in "General tasks and demands". In contrast, *performance *(right) was markedly better than *capacity *(left), as reflected by the lower proportions of severe or complete disability in the "Mobility", "Self-care" and "Domestic life" domains.

## Discussion

The *EDAD 2008 *is not only the most important database on disability among the Spanish population, but is arguably also one of the most complete, periodic, national population approaches to studying disability worldwide. The *EDAD 2008 *incorporated important contributions, such as data for institutionalised persons. Major differences between the *EDAD 2008 *and previous disability surveys in Spain pertain to the former's additional, newly designated target of providing information on functional dependency in order to support planning and funding of the Spanish dependency system. As a first step, this study sought to link the *EDAD 2008 *to an analysis of functional dependency, by ICF Checklist backcoding and score rescaling of *EDAD 2008 *items restricted to the Activity and Participation domains. An important limitation of this exercise is asymmetry, in that it suffers from a lack of crosswalking between *EDAD 2008 *variables and Body Structures and Functions, as well as a neglect of aspects potentially related to ICF contextual factors in general and to Environmental Factors in particular.

Results show that, in practice, ICF domains such as "Learning and applying knowledge", "Major life areas" and "Community, social and civic life" were disregarded, and others, such as "General tasks and demands", were not appropriately covered by posing just a couple of questions. The frequent NDS bias towards activity-level data, described above in the case of United Nations efforts [5], permeates the *EDAD 2008*. Hence, ICF domain restriction, score rescaling and exclusion of institutionalised persons in age-specific counts may seriously limit the interpretability and international comparability of prevalence figures in respect of both *capacity *and *performance*, whether for single ICF domains or individual total ICF score indices. In addition, much remains to be improved to ensure a homogeneous and complete data-contribution from institutionalised persons in the country's different Autonomous Regions (*Comunidades Autónomas*), which currently constitute the main *EDAD 2008 *stakeholders in terms of health and social services.

Once the most relevant limitations have thus been defined, our results can be said to afford a structured, albeit non-validated, view of the prevalence of ICF disability in Spain in 2008, with approximately 12/1000 severely/completely disabled and 24/1000 moderately disabled persons, and the latter registering relatively high proportions of severe/complete disability in the following subscores: "Self-care", 1/3; "Mobility", 3/10; and "Domestic life", 8/10. These proportions were considerably reduced, though not eliminated, by personal or technical aid.

Interpretation and analysis of results may be better suited to comparisons with other ICF measurements in populations. In a Turkish study on the elderly population, Donmetz et al in 2005 [22] reported median scores for persons found to give at least one positive answer in the WHODAS-2. The fact that we cannot compare our results from ICF prevalence measurements of similar ICF categories obtained from total scores or for specific domains illustrates the implications of application or neglect of epidemiological principles and of descriptive and analytical purposes in population research.

Reported age-specific prevalence of ICF moderate disability among the very old in Spain [23] is remarkably similar to figures cited here for community-dwelling residents, e.g., 10.61% for men and 16.47% for women in the 80-84 age group compared to the corresponding values of 10.2% for men and 16.9% for women shown in Figure 1. Despite the fact that the sample in Virués et al's study was certainly not geographically representative of the Spanish population, the similarities are nonetheless striking, suggesting that the external validity of the risk factors of ICF severe/complete disability identified by this study for the Spanish population is high [Virués Ortega J et al. Medical, Environmental and Personal Factors of Disability in the Elderly People in Spain: A Screening Survey based on the International Classification of Functioning (Gaceta Sanitaria 2011, in press)].

Assuming that our figures are accurate, the numbers obtained by us for the severe or complete disability levels would allow the numbers of severely/extremely disabled persons in Spain to be estimated for the first time. These figures, namely, 135,506 men and 245,331 women, constitute figures that are remarkably lower and easier to interpret than those reported in 1999 for persons with, say, several disabilities [11] but may not be comparable. Prevalence of severe disability among 85-89 year-old community-dwelling residents in Gloucester UK, measured by a postal survey using a validated independence scale, was 13.4% for men and 20.9% for women, approximately double that found for severe/complete capacity in our study [24]. Similar differences were seen in other sex- and age-groups. It is possible that, despite limited comparability, our figures constitute undercounts, an effect reinforced by the impact of assigning 1 to items where data were missing, something that particularly affected men in "Domestic life" and women in outdoor "Mobility".

Thanks to the large sample size, the value our results is almost unique when it comes to describing disability patterns by ICF domain. The higher prevalence registered for women and for "Domestic Life", "Mobility" and "Self-care" replicate similar patterns observed for extremely aged Spanish [23]; and for elderly Turkish subjects, with mean scores when disabled of 50.5, 40.6 and 34.2 respectively, and a total WHODAS-2 score of 23.0 [22].

The fact that the above-mentioned domains with higher prevalence correspond to domains in which prevalence of disability decreases when measured by *performance*, i.e., with personal or technical aid, fits expected changes expected from the ICF framework. The considerable modification of patterns in the moderately disabled group might indicate that such groups constitute a target for provision of services, in cases where severe/extreme disability can be reduced by personal or technical aid. The large size of this group renders it even more relevant for policy-making and points to a large population segment of the Spanish population which, in addition to the approximately 500,000 severely/completely disabled, encompasses more than 50% of the 1,000,000 persons with ICF moderate disability who could potentially benefit from personal aid if available. Since the *EDAD 2008 *furnishes data on time devoted by caregivers and an assessment of the shortfall, Figure 4 may provide some clues for estimating the need -both met and unmet- for help from other persons, and for building an index of such help in terms of work time, e.g., hours/week. Unfortunately, the lack of availability of data for domains such as social participation, limits the usefulness of the *EDAD 2008 *when it comes to estimating the need for help to improve social participation in, e.g., work, leisure or economy. This domain may be particularly relevant for persons affected by non-somatic ailments, such as psychiatric patients whose disability may have been underrepresented in the *EDAD 2008 *due to formulation, inasmuch as it documents occupational status but not the barriers encountered in overcoming restricted access to work. In brief, it would appear that, taking ICF categories as the point of departure, the *EDAD 2008 *may provide a basis for estimating the prevalence of need for personal help, with it being more difficult to address the need for social support required to improve social participation in, e.g., work, leisure or economy.

Focusing prevalence on disease status, the required epidemiological dimension of services research is well recognised. Nevertheless, an ICF-based approach to the design of National Disability Surveys might be difficult to reconcile with usability of an ICF "which does not classify people" despite "ICF classifies health-related states" (quotation marked fragments obtained from ICF ref 1, page 8). Our results may point towards a role for an epidemiological approach which reinforces the concept of functional dependence, by minimising the visualisation of severe mental disorders with sparse limitations of basic ADL and their need of social support. A description of the differences between functional dependency and disability, and the particularities of the Spanish approach to dependency in the context of the ICF has recently been published [25]. The need for bridging and knowledge transfer in the field of disabilities and ageing, possibly related to certain content discrepancies between the ICF and *EDAD 2008*, has been highlighted [3]. It is not known whether differences between the ICF and *EDAD 2008 *evidenced in Table 1 and the limitations of our approach rely on the lack of bridging between the Spanish group who drew up the survey and the existing international ICF-WHO groups (e.g., ICF/FDRG, ICF Spanish Network on Disabilities, and the above-mentioned experiences of the Washington City Group and Ireland) or on deeper ICF constraints discussed by Salvador-Carulla and others [25].

To sum up, the Spanish *EDAD 2008 *represents a powerful initiative and considerable limitations in terms of applying the ICF to questionnaire design and epidemiological analysis of disability. However, the *EDAD 2008 *can be said to be a useful tool for describing the prevalence of disability and, potentially, for estimating functional dependence for planning aid and services, taking social and geographical differences into account. Approximately 500,000 to 1,000,000 persons present with ICF levels of severe/complete and moderate disability, respectively. In terms of magnitude, such figures fit reported age-specific prevalence data for disability levels obtained from the WHODAS-2 for the very old in Spain, and match the higher difficulty pattern in mobility, domestic life and self-care domains.

## Competing interests

The authors declare that they have no competing interests.

## Financial support

This study was partially supported by the Consortium for Biomedical Research in Neurodegenerative Diseases (*Centro de Investigación Biomédica en Red sobre Enfermedades Neurodegenerativas *- *CIBERNED*), Spanish Health Research Fund (*Fondo de Investigaciones Sanitarias*) project PI06/1098 and Farasdués Foundation.

## Pre-publication history

The pre-publication history for this paper can be accessed here:

http://www.biomedcentral.com/1471-2458/11/897/prepub
